# Consumption of a Western-Style Diet Modulates the Response of the Murine Gut Microbiome to Ciprofloxacin

**DOI:** 10.1128/mSystems.00317-20

**Published:** 2020-07-28

**Authors:** Damien J. Cabral, Jenna I. Wurster, Benjamin J. Korry, Swathi Penumutchu, Peter Belenky

**Affiliations:** a Department of Molecular Microbiology and Immunology, Brown University, Providence, Rhode Island, USA; Duke University

**Keywords:** diet, antibiotics, metagenomics, metatranscriptomics, dysbiosis

## Abstract

Dietary composition and antibiotic use have major impacts on the structure and function of the gut microbiome, often resulting in dysbiosis. Despite this, little research has been done to explore the role of host diet as a determinant of antibiotic-induced microbiome disruption. Here, we utilize a multi-omic approach to characterize the impact of Western-style diet consumption on ciprofloxacin-induced changes to gut microbiome structure and transcriptional activity. We found that Western diet consumption dramatically increased *Bacteroide*s abundances and shifted the community toward the metabolism of simple sugars and mucus glycoproteins. Mice consuming a Western-style diet experienced a greater expansion of *Firmicutes* following ciprofloxacin treatment than those eating a control diet. Transcriptionally, we found that ciprofloxacin reduced the abundance of tricarboxylic acid (TCA) cycle transcripts on both diets, suggesting that carbon metabolism plays a key role in the response of the gut microbiome to this antibiotic. Despite this, we observed extensive diet-dependent differences in the impact of ciprofloxacin on microbiota function. In particular, at the whole-community level we detected an increase in starch degradation, glycolysis, and pyruvate fermentation following antibiotic treatment in mice on the Western diet, which we did not observe in mice on the control diet. Similarly, we observed diet-specific changes in the transcriptional activity of two important commensal bacteria, Akkermansia muciniphila and Bacteroides thetaiotaomicron, involving diverse cellular processes such as nutrient acquisition, stress responses, and capsular polysaccharide (CPS) biosynthesis. These findings demonstrate that host diet plays a role in determining the impacts of ciprofloxacin on microbiome composition and microbiome function.

**IMPORTANCE** Due to the growing incidence of disorders related to antibiotic-induced dysbiosis, it is essential to determine how our “Western”-style diet impacts the response of the microbiome to antibiotics. While diet and antibiotics have profound impacts on gut microbiome composition, little work has been done to examine their combined effects. Previous work has shown that nutrient availability, influenced by diet, plays an important role in determining the extent of antibiotic-induced disruption to the gut microbiome. Thus, we hypothesize that the Western diet will shift microbiota metabolism toward simple sugar and mucus degradation and away from polysaccharide utilization. Because of bacterial metabolism’s critical role in antibiotic susceptibility, this change in baseline metabolism will impact how the structure and function of the microbiome are impacted by ciprofloxacin exposure. Understanding how diet modulates antibiotic-induced microbiome disruption will allow for the development of dietary interventions that can alleviate many of the microbiome-dependent complications of antibiotic treatment.

## Abstract (precis)

Due to the growing incidence of disorders related to antibiotic-induced dysbiosis, it is essential to determine how our “Western”-style diet impacts the response of the microbiome to antibiotics. While diet and antibiotics have profound impacts on gut microbiome composition, little work has been done to examine their combined effects. Previous work has shown that nutrient availability, influenced by diet, plays an important role in determining the extent of antibiotic-induced disruption to the gut microbiome. Thus, we hypothesize that the Western diet will shift microbiota metabolism toward simple sugar and mucus degradation and away from polysaccharide utilization. Because of bacterial metabolism’s critical role in antibiotic susceptibility, this change in baseline metabolism will impact how the structure and function of the microbiome are impacted by ciprofloxacin exposure. Understanding how diet modulates antibiotic-induced microbiome disruption will allow for the development of dietary interventions that can alleviate many of the microbiome-dependent complications of antibiotic treatment.

## INTRODUCTION

The gut microbiome includes the trillions of largely commensal bacteria, archaea, and fungi that inhabit the gastrointestinal tract (1–3). These communities play an important role in numerous biological processes such as digestion, neurological development, colonization resistance, and immune function (4–17). Consequently, it is unsurprising that disruption of microbial homeostasis, termed dysbiosis, has numerous harmful impacts to the host. The gut microbiome is highly sensitive to perturbations such as broad-spectrum antibiotic usage. Within hours of treatment, antibiotics induce dramatic reductions in both bacterial load and diversity within the microbiome, both of which are common indicators of dysbiosis (18, 19).

While compositional changes are typically transient and recover following the cessation of a perturbation, oftentimes the structure and diversity of the microbiota never return to their original levels. The resulting dysbiosis often has numerous acute and chronic impacts on host health. In the case of antibiotic usage, this may increase the risk of infection with opportunistic fungal and bacterial pathogens by reducing colonization resistance (1, 4, 5, 17, 20–24). Most notably, broad-spectrum antibiotic treatment is a major risk factor in Clostridioides difficile infection (20, 22, 25, 26). Persistent dysbiosis is correlated with many chronic conditions with considerable morbidity and mortality, such as asthma, obesity, and inflammatory bowel disease (6–9, 11, 13, 14, 17, 26).

Interestingly, antibiotic-induced disruption of the microbiome may be influenced by the metabolic environment of the gut. A large body of *in vitro* data indicates that the rate of metabolic activity for bacteria correlates positively with antimicrobial susceptibility, such that metabolically active, ATP-producing processes such as respiration promote toxicity, whereas less efficient or quiescent metabolism induces tolerance (27–29). A similar trend is observed in the context of bacteria responding to antibiotics in the gut microbiome, where nutrient availability and bacterial metabolism are closely linked to host diet. Recent work has demonstrated that antibiotic exposure changes both the composition of the gut microbiome and its metabolic capacity, such that the surviving microbiome is overall less metabolically active (19). Further, amoxicillin treatment was shown to increase the expression of polysaccharide utilization genes, while simultaneously decreasing the abundance of transcripts involved in simple sugar utilization (19). Reflecting these changes, amoxicillin also decreased the total concentration of glucose within the ceca of mice (19). These transcriptional changes have significant impacts on the response of specific bacteria to the treatment. In the case of Bacteroides thetaiotaomicron, polysaccharide utilization promoted tolerance to amoxicillin, and simple sugar utilization increased toxicity. Accordingly, the response of the microbiota to antibiotics can be impacted by dietary nutrient modulation (30). For example, Cabral et al. found that glucose supplementation impacts the response of the total community and reduces the absolute abundance of bacteria, particularly B. thetaiotaomicron, following amoxicillin treatment in mice (19). Together these findings suggest that dietary composition may act as an additional perturbation that drives the severity of the microbiome’s response to antibiotic treatment.

Dietary composition is known to have a profound impact on microbiome diversity and overall gut health (31–37). Diets high in fat and simple sugars, typically referred to as “Western” diets, have been associated with a number of negative health states including obesity, diabetes mellitus, allergies, and inflammatory bowel disease (36–46). Such diets have very low levels of microbiota-accessible carbohydrates (MACs), which are typically found in complex plant polysaccharides and are indigestible and unabsorbable by the host (40, 44, 47–49). MACs are typically fermented by the colonic microbiota to produce short-chain fatty acids (SCFAs), which play important roles in regulating energy homeostasis and inflammation within the host (40, 45, 50–55). High-MAC diets have also been shown to increase microbial diversity, a classic benchmark for gut microbiota health. Conversely, low-MAC diets are known to reduce both microbiome diversity and SCFA production (44, 46, 49, 56). MAC starvation enriches for muciniphilic microbes that are capable of degrading the mucosal lining of the gut, such as Akkermansia muciniphila (40, 42, 48, 57). Degradation of the mucosal layer over time may result in compromised gut barrier function and lead to increased inflammation, colitis, and susceptibility to infection by enteric pathogens (57).

Individually, antibiotic usage and the consumption of Western-style diets are known to negatively impact the microbiota, impacting host health. Despite this, little work has explored the impact of diet on the response of the microbiota to antibiotics. Previous work has suggested that dietary composition may play an important role in determining the extent of antibiotic-induced microbiome disruption (19). Thus, we hypothesize that the consumption of a Western-style diet will significantly modify the metabolic activity of the microbiome toward simple sugar and mucus glycoprotein degradation rather than dietary polysaccharide utilization. This will be characterized by differential utilization of carbohydrate-active enzymes (CAZymes) along with changes in respiratory activity and central carbon metabolism. Given that respiratory activity plays a key role in drug susceptibility *in vitro*, when this community is treated with a bactericidal antibiotic like ciprofloxacin, its compositional and functional responses to the drug would be different due to the altered metabolic state. Overall, we anticipate that the diet-related metabolic state of the microbiome before treatment will have a larger impact on drug disruption than the metabolic changes that are induced during the drug exposure. In this study, we use a combined metagenomic and metatranscriptomic approach to characterize the impact of a Western-style diet on the taxonomic and functional disruption of the microbiome during ciprofloxacin treatment. Using shotgun metagenomics, we found that ciprofloxacin elicited differential impacts on community composition in mice at both the phylum and species level in a diet-dependent manner. Using metatranscriptomics, we observed that consumption of a Western diet induced profound transcriptional changes within the gut microbiomes of mice. Furthermore, consumption of this diet modulated the transcriptional response of these communities to antibiotic treatment. Specifically, dietary composition had a major impact on the abundance of transcripts containing key metabolic genes. Lastly, we were able to detect unique species-specific transcriptional changes in response to both diet and ciprofloxacin treatment in two important commensal bacteria, *A. muciniphila* and B. thetaiotaomicron.

## RESULTS

**FIG 1:**
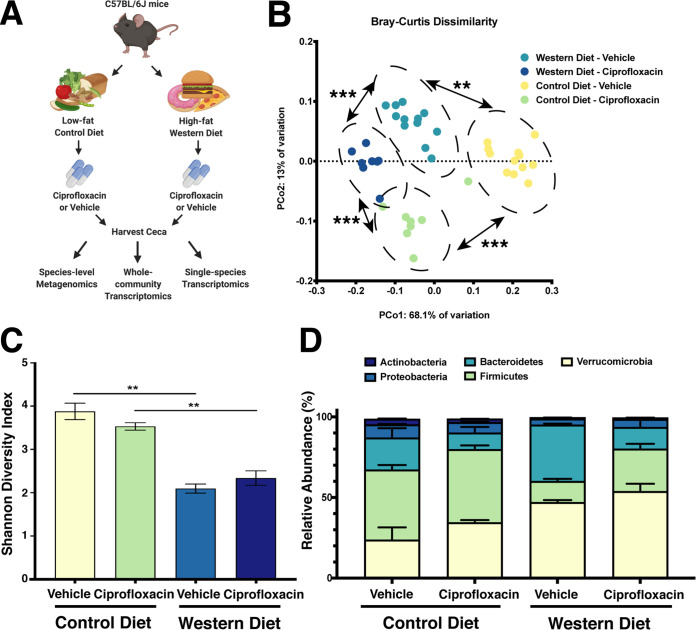
Impact of diet and ciprofloxacin administration on murine gut microbiome composition. (A) Experimental workflow used in this study. Figure was created with BioRender.com (BioRender, Toronto, Canada). (B) Principal Coordinate Analysis of experimental groups as measured by Bray-Curtis dissimilarity of 16S rRNA amplicons. (**, *P* < 0.01; ***, *P* < 0.001, permutational ANOVA). (C) Alpha diversity of experimental groups as measured by the Shannon diversity index. Data are represented as mean ± standard error of the mean (SEM) (**, *P* < 0.01, Welch ANOVA with Dunnett T3 test for multiple hypothesis testing). (D) Stacked bar plot of the five most abundant bacterial phyla in our data set. Data are represented as mean ± SEM for each phylum. For 16S rRNA amplicons, *n* = 8 to 12. For metagenomics, *n* = 4.

To determine the impact of dietary composition and antibiotic exposure on the structure and function of the murine gut microbiome, female C57BL/6J mice were randomly assigned to either a high-fat, high-sugar “Western”-style (Western) diet or a low-fat control diet for 7 days in multiple cages. At this point, mice from each diet were again randomly split between ciprofloxacin and vehicle control groups and treated for 24 h in multiple cages (*n* = 8 to 12 per group). Previously it has been shown that 24 h of ciprofloxacin treatment was sufficient to induce changes in community structure and transcriptional activity (19). This time frame also allowed for profiling the acute response of the microbiota to ciprofloxacin exposure, rather than characterizing a post-antibiotic state of equilibrium. Following treatment, the mice were sacrificed to harvest their cecal contents for taxonomic profiling and transcriptional analysis (Fig. 1A). Overall, we found that diet and ciprofloxacin treatment had a significant impact on gut microbiome structure (Fig. 1B to D; see also Fig. S1 in the supplemental material).

10.1128/mSystems.00317-20.1 FIG S1 Dietary composition and antibiotic treatment impact the diversity of the gut microbiome. (A) Alpha diversity of experimental groups as measured by the Shannon diversity index of 16S rRNA amplicons. Data are represented as mean ± standard error of the mean (SEM) (*, *P* < 0.05; **, *P* < 0.01; ***, *P* < 0.001; Welch ANOVA with Dunnett T3 test for multiple hypothesis testing). (B) Stacked bar plot of the 10 most abundant bacterial species in our data set. Data are represented as mean ± SEM for each species. For 16S rRNA amplicons, *n* = 8 to 12. For metagenomics, *n* = 4. Download FIG S1, PDF file, 0.2 MB. Copyright © 2020 Cabral et al. 2020 Cabral et al. This content is distributed under the terms of the Creative Commons Attribution 4.0 International license.

We first assessed the effects that diet and ciprofloxacin have on the diversity of the gut microbiome using 16S rRNA sequencing. Mice consuming the Western diet had significantly less diverse gut microbiomes than those fed the control diet (Fig. S1A). Interestingly, we also observed that the Western diet was associated with a reduction in alpha diversity during ciprofloxacin treatment (Fig. S1A). Next, we performed Principal Coordinate Analysis (PCoA) using Bray-Curtis dissimilarity paired with permutational multivariate analysis of variance (PERMANOVA) to profile the degree of dissimilarity between our samples and the significance of this distance. Our samples formed four distinct clusters driven by both diet and ciprofloxacin treatment (Fig. 1B).

Due to the limited phylogenetic resolution provided by 16S rRNA sequencing and inability to provide functional information about sequenced communities, we opted to perform shotgun metagenomic and metatranscriptomic analyses on a subset of our samples, representing mice from multiple cages (*n* = 4 per treatment group) (19, 58–61). Interestingly, we observed that Western diet consumption reduced community diversity while ciprofloxacin did not have a statistically significant impact on the alpha diversity of the community (Fig. 1C). However, the metagenomic data exhibited a similar trend in unique taxonomic structures being associated with each treatment group, supporting a model wherein diet and antibiotic treatment are distinct perturbations (Fig. 1D). However, to evaluate if diet modifies the response to ciprofloxacin, we had to untangle diet-induced changes from antibiotic-induced changes. First, we characterized the impact of the Western diet consumption.

### Consumption of a Western diet modifies the metabolic activity of the microbiome.

**FIG 2:**
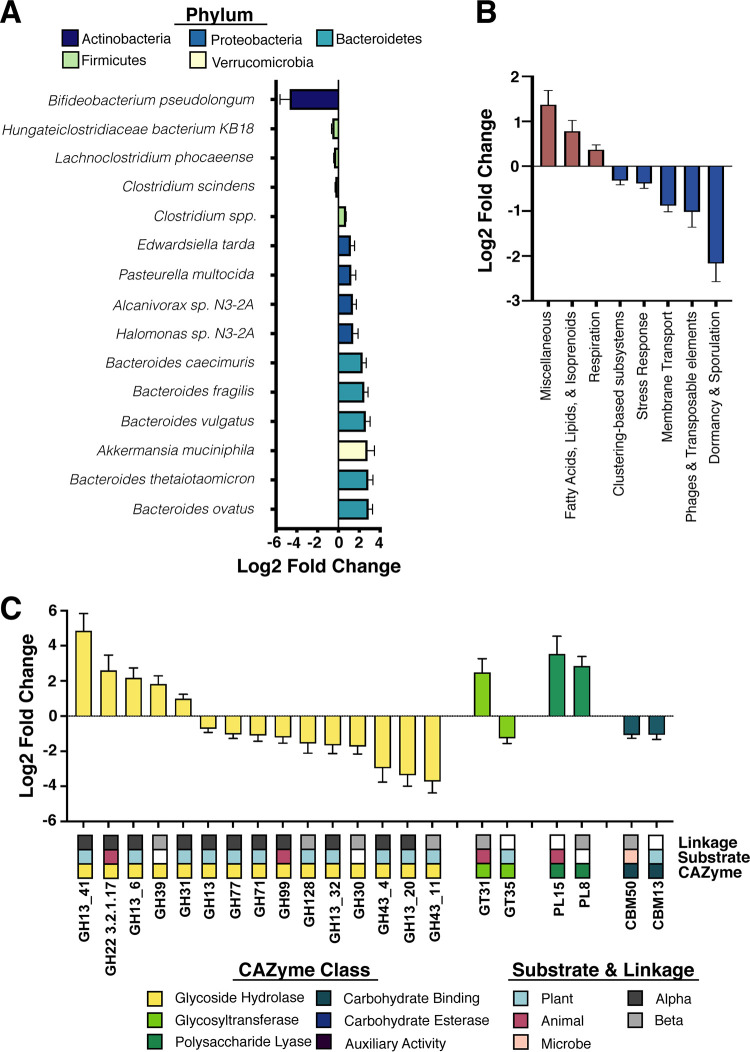
Consumption of a Western diet induces broad taxonomic and transcriptional changes at the community level. (A) Differentially abundant (Benjamini-Hochberg-adjusted *P* value < 0.05) bacterial species (within the 45 most abundant taxa) as detected in mice consuming the Western diet. Data are represented as log_2_ fold change relative to control diet ± standard error. Bar color and top legend denote phylum-level taxonomic classification (yellow, *Verrucomicrobia*; green, *Firmicutes*; teal, *Bacteroidetes*; blue, *Proteobacteria*; navy, *Actinobacteria*). See Data Set S1 for full results. (B) Differentially expressed (Benjamini-Hochberg-adjusted *P* value < 0.05) level 1 SEED subsystems in the murine cecal metatranscriptome of mice consuming the Western diet. Data are represented as log_2_ fold change relative to control diet ± standard error. Only features with a base mean of ≥100 were plotted. See Data Set S3 for full results. (C) Differentially expressed (Benjamini-Hochberg-adjusted *P* value < 0.05) CAZyme transcripts in the murine cecal metatranscriptome in mice consuming the Western diet. Data are represented as log_2_ fold change relative to control diet ± standard error. CAZyme class (yellow, glycoside hydrolase; lime, glycosyltransferase; green, polysaccharide lyase; teal, carbohydrate binding modules; blue, carbohydrate esterase; purple, auxiliary activity), source of the target substrate (blue, plant derived; magenta, animal derived; peach, microbially derived), and linkages targeted by the CAZyme (dark gray, alpha; light gray, beta) are listed below the data and color coded. White values represent either a lack of singular substrate/linkage or a lack of enough information available to make a definitive call. See Data Set S5 for full results. For all analyses, *n* = 4.

Mice fed a Western diet displayed elevated levels of the phyla *Verrucomicrobia* and *Bacteroidetes* and a reduction of *Firmicutes* (Fig. 1D). At the species level, these shifts appear to be largely driven by an expansion of members of the *Bacteroides* genus (Fig. 2A, Fig. S1B, and Data Set S1). Additionally, the Western diet-fed mice displayed an elevated abundance of several species from the *Proteobacteria* phylum, suggestive of dysbiosis (62). Two important bacterial species found in the gut microbiomes of both mice and humans, B. thetaiotaomicron and *A. muciniphila*, were observed at significantly elevated levels in the mice fed a Western diet (Fig. 2A and Fig. S1B). Notably, both species are known to utilize host-produced mucins; thus, this observation is consistent with earlier studies suggesting that the consumption of a low-MAC Western diet enriches for muciniphilic bacteria (40, 42, 48).

10.1128/mSystems.00317-20.4 DATA SET S1 Full DESeq2 results of differential abundance testing of top 45 species detected by shotgun metagenomics. (Sheet 1) Differential abundance testing of the impact of Western diet (WD) consumption on the abundance of the top 45 bacterial species detected in our data set. Log_2_ fold change values were calculated relative to control diet samples. (Sheet 2) Differential abundance testing of the impact of ciprofloxacin treatment on the abundance of the top 45 bacterial species in mice consuming the Western diet (WD). Log_2_ fold change values were calculated relative to vehicle-treated samples on the WD. (Sheet 3) Differential abundance testing of the impact of ciprofloxacin treatment on the abundance of the top 45 bacterial species in mice consuming the control diet (NC). Log_2_ fold change values were calculated relative to vehicle-treated samples on the NC. (Sheet 4) Interaction term analysis generated by DESeq2 for the impact of host diet consumption on changes in species abundance following ciprofloxacin therapy. Log_2_ fold change values were calculated relative to vehicle-treated samples on the NC. Download Data Set S1, XLS file, 0.05 MB. Copyright © 2020 Cabral et al. 2020 Cabral et al. This content is distributed under the terms of the Creative Commons Attribution 4.0 International license.

10.1128/mSystems.00317-20.6 DATA SET S3 Full DESeq2 results of SEED subsystem abundance generated by SAMSA2. (Sheet 9) Differential abundance testing of the impact of Western diet (WD) consumption on the abundance of SEED subsystems in the murine cecal metatranscriptome. Log_2_ fold change values were calculated relative to control diet samples. (Sheet 10) Differential abundance testing of the impact of ciprofloxacin treatment on the abundance of SEED subsystems in the murine cecal metatranscriptome in animals consuming the Western diet (WD). Log_2_ fold change values were calculated relative to vehicle-treated samples on the WD. (Sheet 11) Differential abundance testing of the impact of ciprofloxacin treatment on the abundance of SEED subsystems in the murine cecal metatranscriptome in animals consuming the control diet (NC). Log_2_ fold change values were calculated relative to vehicle-treated samples on the NC. Download Data Set S3, XLS file, 0.04 MB. Copyright © 2020 Cabral et al. 2020 Cabral et al. This content is distributed under the terms of the Creative Commons Attribution 4.0 International license.

10.1128/mSystems.00317-20.8 DATA SET S5 Full DESeq2 results of CAZyme transcript abundance generated by SAMSA2. (Sheet 16) Differential abundance testing of the impact of Western diet (WD) consumption on the abundance of CAZyme transcripts in the murine cecal metatranscriptome. Log_2_ fold change values were calculated relative to control diet samples. (Sheet 17) Differential abundance testing of the impact of ciprofloxacin treatment on the abundance of CAZyme transcripts in the murine cecal metatranscriptome in animals consuming the Western diet (WD). Log_2_ fold change values were calculated relative to vehicle-treated samples on the WD table. (Sheet 18) Differential abundance testing of the impact of ciprofloxacin treatment on the abundance of CAZyme transcripts in the murine cecal metatranscriptome in animals consuming the control diet (NC). Log_2_ fold change values were calculated relative to vehicle-treated samples on the NC. (Sheet 19) Interaction term analysis generated by DESeq2 for the impact of host diet consumption on changes in CAZyme transcripts abundance following ciprofloxacin therapy. Log_2_ fold change values were calculated relative to vehicle-treated samples on the NC. Download Data Set S5, XLS file, 0.4 MB. Copyright © 2020 Cabral et al. 2020 Cabral et al. This content is distributed under the terms of the Creative Commons Attribution 4.0 International license.

Given this expansion, we anticipated that the transcriptional activity of these communities would exhibit an increased capacity for mucus degradation and simple sugar utilization. Due to the potential limitations of using a single pipeline, we analyzed our metatranscriptomic data set with SAMSA2 in parallel with HUMAnN2 (63, 64). The SAMSA2 pipeline generates unnormalized transcript abundances and thus is representative of overall transcript levels (63). SAMSA2 is advantageous in its capacity for annotation against multiple databases and enables differential abundance testing of individual transcripts in addition to pathway- and subsystem-level analysis (63). Conversely, the HUMAnN2 pipeline normalizes the abundance of RNA transcripts against their corresponding gene abundance in the metagenomic data set, thus normalizing for differences in community composition between experimental groups and facilitating comparisons of metabolic pathway expression at the whole-community level (64). When paired, these pipelines facilitate a more robust examination of microbiome transcriptional activity.

We observed an increased abundance of transcripts related to respiration at the SEED subsystem level in the microbiota of the mice consuming the Western diet, which was mirrored in our HUMAnN2 data set as increased tricarboxylic acid (TCA) cycle expression (Fig. 2B, Fig. S2A, and Data Sets S2 and S3). The Western diet-fed mouse microbiota also displayed increased abundance of transcripts involving fatty acid metabolism and terpenoid biosynthesis, likely reflecting altered nutrient availability and increased respiratory activity, respectively (Fig. 2B and Data Set S3) (65, 66). Interestingly, we also detected large increases in the abundance of two different sialidase transcripts, which play a key role in the utilization of host-produced mucins (Fig. S2B and Data Set S4) (67). While other studies have shown that the consumption of a Western diet enriches for muciniphilic taxa, this observation suggests that this diet also increases transcriptional activity related to mucin degradation within the microbiome (40, 42).

10.1128/mSystems.00317-20.2 FIG S2 Consumption of a Western diet induces broad taxonomic and transcriptional changes at the community and species level. (A) Linear discriminant analysis (LDA) of MetaCyc pathways that were differentially associated with either the control or Western diet. Bar size indicates LDA score, and color indicates the experimental group (blue, Western diet; yellow, control diet) that a MetaCyc pathway was significantly associated with. All LDA scores were generated using LEfSe on unstratified pathway outputs from HUMAnN2. For full pathway names and statistics, see Data Set S2. (B) Volcano plot of the metatranscriptomic profile of the murine cecal microbiome in vehicle-treated mice consuming Western diet. Data were generated by aligning metatranscriptomic reads to RefSeq using SAMSA2 and analyzing using DESeq2. Points in purple represent transcripts for which a statistically significant change in expression was detected (Benjamini-Hochberg-adjusted *P* value < 0.05). Select genes of interest are labeled. See Data Set S4 for full results. For all analyses, *n* = 4. Download FIG S2, PDF file, 0.5 MB. Copyright © 2020 Cabral et al. 2020 Cabral et al. This content is distributed under the terms of the Creative Commons Attribution 4.0 International license.

10.1128/mSystems.00317-20.7 DATA SET S4 Full DESeq2 results of RefSeq transcript abundance generated by SAMSA2. (Sheet 12) Differential abundance testing of the impact of Western diet (WD) consumption on the abundance of RefSeq transcripts in the murine cecal metatranscriptome. Log_2_ fold change values were calculated relative to control diet samples. (Sheet 13) Differential abundance testing of the impact of ciprofloxacin treatment on the abundance of RefSeq transcripts in the murine cecal metatranscriptome in animals consuming the Western diet (WD). Log_2_ fold change values were calculated relative to vehicle-treated samples on the WD. (Sheet 14) Differential abundance testing of the impact of ciprofloxacin treatment on the abundance of RefSeq transcripts in the murine cecal metatranscriptome in animals consuming the control diet (NC). Log_2_ fold change values were calculated relative to vehicle-treated samples on the NC. (Sheet 15) Interaction term analysis generated by DESeq2 for the impact of host diet consumption on changes in RefSeq transcripts abundance following ciprofloxacin therapy. Log_2_ fold change values were calculated relative to vehicle-treated samples on the NC. Download Data Set S4, XLS file, 5.6 MB. Copyright © 2020 Cabral et al. 2020 Cabral et al. This content is distributed under the terms of the Creative Commons Attribution 4.0 International license.

Additionally, the Western diet-fed mouse microbiota had reduced expression of nucleotide biosynthesis, glycolysis, gluconeogenesis, starch degradation, and pyruvate fermentation compared to control diet-fed mice (Fig. S2A and Data Set S2). We also observed relative reduction in the expression of the *Bifidobacterium* shunt, which is known to play a role in SCFA production and may provide mechanistic insight into the reduced SCFA levels observed on the Western diet in other studies (Fig. S2A and Data Set S2) (40, 51).

10.1128/mSystems.00317-20.5 DATA SET S2 Full LEfSe results from the analysis of MetaCyc pathway abundance generated by HUMAnN2. “Class” denotes the experimental group that a particular pathway was associated with. (Sheet 5) LEfSe analysis of all experimental groups. (Sheet 6) Pairwise LEfSe analysis of vehicle-treated samples from mice consuming either the Western (WD) or control (NC) diet. (Sheet 7) Pairwise LEfSe analysis of ciprofloxacin- and vehicle-treated samples from mice consuming the control diet (NC). (Sheet 8) Pairwise LEfSe analysis of ciprofloxacin- and vehicle-treated samples from mice consuming the Western diet (WD). Download Data Set S2, XLS file, 0.1 MB. Copyright © 2020 Cabral et al. 2020 Cabral et al. This content is distributed under the terms of the Creative Commons Attribution 4.0 International license.

Examination of CAZyme activity provided further evidence of significant transcriptional reprogramming in response to diet. Specifically, we observed that Western diet consumption decreased transcript abundances of multiple enzymes involved in polysaccharide breakdown (Fig. 2C and Data Set S5) (68–71). Simultaneously, there was a significant increase in α-amylases, lysozyme C, and α-lactalbumin breakdown (Fig. 2C and Data Set S5) (72, 73). Given the content of the Western diet, a shift toward utilization of these carbon sources was not unexpected. However, the robust loss of complex polysaccharide breakdown was surprising and complements the SEED and HUMAnN2 data sets. Together these data suggest that Western diet alone is sufficient to restructure the metabolic activity of the gut microbiome, due to significant changes in nutrient availability.

### Ciprofloxacin elicits unique shifts in gene expression on Western and control diets.

**FIG 3:**
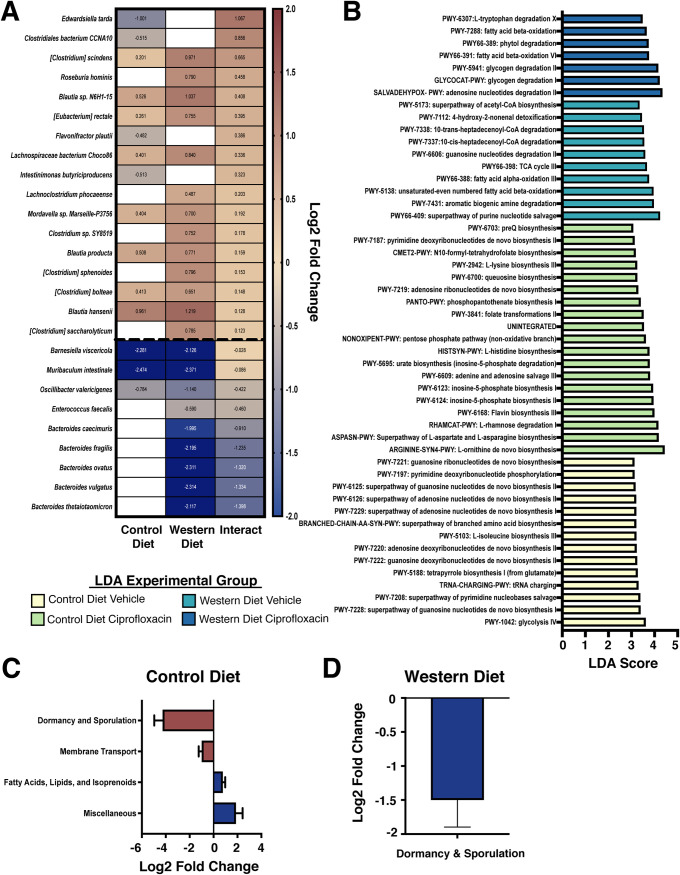
Ciprofloxacin elicits unique shifts in gene expression on Western and control diets at the community level. (A) Heatmap of the change in abundance of the top 45 bacterial species in response to ciprofloxacin on control and Western diets. The Interact column represents the interaction term generated by DESeq2, denoting the impact of diet on the change in abundance of each species to ciprofloxacin compared to vehicle control. Cell color denotes log_2_ fold change of a particular species in response to ciprofloxacin (white represents failure to meet statistical significance: Benjamini-Hochberg-adjusted *P* value < 0.05). Heatmap rows were sorted by interaction term value from highest to lowest, and taxa with no differential abundance (failure to meet statistical significance) in either group were removed. See Data Set S1 for full DESeq2 results. (B) Linear discriminant analysis (LDA) of MetaCyc pathways that were differentially associated with each experimental group. Bar size indicates LDA score, and color indicates the experimental group that a MetaCyc pathway was significantly associated with. All LDA scores were generated using LEfSe on unstratified pathway outputs from HUMAnN2. For full pathway names and statistics, see Data Set S2. (C) Differentially expressed (Benjamini-Hochberg-adjusted *P* value < 0.05) level 1 SEED subsystems in the murine cecal metatranscriptome after ciprofloxacin treatment in mice consuming the control diet. Data are represented as log_2_ fold change relative to vehicle controls ± standard error. Only features with a base mean of ≥100 were plotted. See Data Set S3 for full results. (D) Differentially expressed (Benjamini-Hochberg-adjusted *P* value < 0.05) level 1 SEED subsystems in the murine cecal metatranscriptome after ciprofloxacin treatment in mice consuming the Western diet. Data are represented as log_2_ fold change relative to vehicle controls ± standard error. Only features with a base mean of ≥100 were plotted. See Data Set S3 for full results. For all analyses, *n* = 4.

Given the significant body of literature that links microbial metabolism with antimicrobial susceptibility both *in vitro* and within the microbiome, we hypothesized that the metabolic restructuring induced by the Western diet would result in differential susceptibility to ciprofloxacin (19, 27–29). Although ciprofloxacin did not induce a significant reduction in alpha diversity in the time frame tested, we found that diet drove differential community composition following antibiotic exposure (Fig. 1C and D). At the phylum level, we observed a significant expansion in the relative abundance of *Firmicutes* following ciprofloxacin treatment on the Western diet (adjusted *P* value = 0.0388) but not on the control diet (adjusted *P* value = 0.8718) (Fig. 1D and Fig. S1B). To determine which species displayed a differential response to ciprofloxacin on the Western and control diets, we utilized DESeq2 to analyze the interaction between diet and antibiotic treatment to determine which species displayed differential responses to ciprofloxacin between the diets (74). While most species responded similarly to ciprofloxacin therapy on both diets, there were several notable exceptions. For example, the expansion of several *Clostridium* species (such as Clostridium saccharolyticum, Clostridium sphenoides, and Clostridium scindens) following ciprofloxacin was higher on the Western diet than the control (positive interaction values, Fig. 3A and Data Set S1). Conversely, the reduction of several *Bacteroides* species following antibiotic treatment tended to be exacerbated on the Western diet (negative interaction values, Fig. 3A and Data Set S1).

We detected clear differences in ciprofloxacin susceptibility between the two diets and hypothesized that diet-induced differences in metabolism would both alter susceptibility and be reflected in unique transcriptional signatures. An all-by-all comparison of experimental groups demonstrated that the microbiota of Western diet-consuming mice displayed elevated expression of TCA cycle and fatty acid degradation pathways in both vehicle and ciprofloxacin treatments, likely reflective of the increased fat and sugar content of this diet (Fig. 3B and Data Set S2). Additionally, we found elevated expression of glycogen degradation genes that was specific to Western diet-fed mice receiving ciprofloxacin (Fig. 3B and Data Set S2). Conversely, the microbiota of control diet-consuming mice had elevated expression of amino acid biosynthesis pathways (isoleucine, aspartate, asparagine, lysine, and histidine) regardless of antibiotic treatment (Fig. 3B and Data Set S2). We also observed elevated levels of several different nucleotide biosynthesis pathways in the vehicle-treated control diet mice while the Western diet mice displayed elevated levels of adenosine and guanosine nucleotide degradation (Fig. 3B and Data Set S2). Overall, these data support that our experimental groups could be characterized by unique transcriptional signatures.

We found key differences in the overall transcriptional profiles in response to ciprofloxacin on each diet. On the Western diet, ciprofloxacin treatment was associated with an increased abundance of transcripts from glycogen and starch degradation, glycolysis, and pyruvate fermentation (Fig. S3C and Data Set S2). Notably, the expression of glycogen degradation was elevated in vehicle-treated samples on the control diet, suggesting that the utilization of this pathway during ciprofloxacin treatment is diet dependent (Fig. S3C and Data Set S2). We observed that TCA cycle expression was reduced in ciprofloxacin-treated mice compared to the vehicle treatment—the lone commonality between diets (Fig. S3C and Data Set 2). Previous work has demonstrated that TCA cycle elevation increases sensitivity to bactericidal antibiotics (27–29, 75). Thus, this result suggests that TCA cycle activity may play a key role in the response of the microbiota to ciprofloxacin treatment *in vivo*, though more work is required to understand its impact.

10.1128/mSystems.00317-20.3 FIG S3 Ciprofloxacin elicits unique shifts in gene expression on Western and control diets at the community and species level. (A) Volcano plot of the metatranscriptomic profile of the murine cecal microbiome in ciprofloxacin-treated mice on the control diet. Data were generated by aligning metatranscriptomic reads to RefSeq using SAMSA2 and analyzing using DESeq2. Points in purple represent transcripts for which a statistically significant change in expression was detected (Benjamini-Hochberg-adjusted *P* value < 0.05). Select genes of interest are labeled. See Data Set S4 for full results. (B) Volcano plot of the metatranscriptomic profile of the murine cecal microbiome in ciprofloxacin-treated mice on the Western diet. Data generation, point labeling, and statistical cutoffs are the same as for panel A. See Data Set S4 for full results. (C) Linear discriminant analysis (LDA) of MetaCyc pathways that were differentially associated with either the control or Western diet during ciprofloxacin treatment. LDA score indicates the experimental group that a MetaCyc pathway was significantly associated with (negative values, vehicle treatment; positive values, ciprofloxacin treatment). All LDA scores were generated using LEfSe on unstratified pathway outputs from HUMAnN2 (white represents failure to meet statistical significance). For full pathway names and statistics, see Data Set S2. (D) Differentially expressed (Benjamini-Hochberg-adjusted *P* value < 0.05) CAZyme transcripts in B. thetaiotaomicron within mice consuming the control diet during ciprofloxacin treatment. Data are represented as log_2_ fold change relative to control diet ± standard error. CAZyme class (yellow, glycoside hydrolase; lime, glycosyltransferase; green, polysaccharide lyase; teal, carbohydrate binding modules; blue, carbohydrate esterase; purple, auxiliary activity), source of the target substrate (blue, plant derived; magenta, animal derived; peach, microbially derived), and linkages targeted by the CAZyme (dark gray, alpha; light gray, beta) are listed to the left of the data and color coded. White values represent either a lack of singular substrate/linkage or a lack of enough information available to make a definitive call. See Data Set S7 for full results. (E) Differentially expressed (Benjamini-Hochberg-adjusted *P* value < 0.05) CAZyme transcripts in B. thetaiotaomicron within mice consuming the Western diet during ciprofloxacin treatment. Data are represented as log_2_ fold change relative to control diet ± standard error. CAZyme class, source of target substrate, and linkages targeted by the CAZyme are listed below the data and color coded as described for panel D. See Data Set S7 for full results. (F) Differentially expressed (Benjamini-Hochberg-adjusted *P* value < 0.05) CAZyme transcripts in *A. muciniphila* within mice consuming either diet during ciprofloxacin treatment. Data are represented as log_2_ fold change relative to control diet ± standard error. CAZyme class, source of target substrate, and linkages targeted by the CAZyme are listed below the data and color coded as described for panel D. See Data Set S7 for full results. For all analyses, *n* = 4. Download FIG S3, PDF file, 0.4 MB. Copyright © 2020 Cabral et al. 2020 Cabral et al. This content is distributed under the terms of the Creative Commons Attribution 4.0 International license.

Interestingly, comparatively few subsystems were changed following ciprofloxacin treatment on either diet (Fig. 3C and D and Data Set S3), suggesting that the pretreatment metabolic state affects the antibiotic response more than the drug-induced transcriptional changes. Most notably, we observed a decrease in transcripts related to dormancy and sporulation in response to ciprofloxacin on both diets (Fig. 3C and D and Data Set S3). A similar finding was observed in a recent study, suggesting that these transcripts may play a key role in the response of the microbiota to this antibiotic (19). Furthermore, ciprofloxacin increased the abundance of sialidase transcripts in mice on the control diet, suggesting that this effect may be exacerbated by antibiotic treatment (Fig. S3A and Data Set S4). Reflecting the overall reduction in sporulation seen at the subsystem level, we found that several sporulation-related transcripts were reduced on the control diet following ciprofloxacin treatment (Fig. S3A and Data Set S4).

We also examined the interaction of diet and antibiotic treatment on transcript abundance within the microbiome. Notably, we found that several sporulation genes were significantly higher on the Western diet than the control following ciprofloxacin treatment (Data Set S4), which was reflected in the SEED subsystem level (Fig. 3C and D). Additionally, transcripts encoding phosphotransferase system (PTS) transporters of various substrates were also found to be higher on the Western diet following ciprofloxacin treatment (Data Set S4). Conversely, Western diet consumption significantly reduced the change in transcript abundance of both pectate lyase and a hemin receptor following ciprofloxacin therapy. Together, these findings demonstrate that dietary composition significantly impacts the transcriptional response of the microbiome to ciprofloxacin.

Recent studies have shown CAZyme activity to be a significant component of the microbiome’s response to antibiotic stress (19). In our study, over 75 CAZymes exhibited differential abundance during ciprofloxacin treatment (Data Set S5). Interestingly, these changes were exclusive to the control diet-fed microbiota, as the Western diet-fed communities displayed no significant difference in CAZyme abundance (Data Set S5). The microbiota of mice on the control diet exhibited increases in CAZymes involved in starch, glycogen, xylose, pectin, rhamnogalacturonan, and arabinofuranose degradation (Data Set S5) (76, 77). Additionally, these communities exhibited a significant increase in trehalose phosphorylase and synthase activity, both of which have been associated with transient antibiotic tolerance in pathogenic species (Data Set S5) (78, 79). Loss of these CAZyme shifts may be directly involved in the increased toxicity of ciprofloxacin on the Western diet; however, more work is required to elucidate the mechanism. These data, in conjunction with our SEED and HUMAnN2 data sets, provide evidence for unique transcriptional signatures during ciprofloxacin challenge that are diet dependent. Overall, this supports a model in which diet-driven differences in baseline metabolism directly impact taxonomic and functional responses to ciprofloxacin treatment.

### Diet and ciprofloxacin alter gene expression within B. thetaiotaomicron and *A. muciniphila*.

Next, we sought to profile how diet and drug treatment impacted the transcriptional response of individual species within the microbiota. In order to have sufficient genome coverage and sequencing depth, we ranked all taxa that were differentially abundant in the Western diet by average RNA reads, further analyzing only those with 500,000 or greater (Data Set S6). With this criterion, we used a previously published pipeline to interrogate the impact of diet and antibiotic treatment on three individual species: B. thetaiotaomicron, *A. muciniphila*, and *C. scindens* (19, 80). We focused on these bacteria because they are known human gut commensals, were found at relatively high levels in all samples analyzed, and were differentially abundant in a diet-dependent manner. Unfortunately, *C. scindens* had relatively few transcriptional changes across all comparisons, and those genes that were differentially regulated were almost exclusively hypothetical proteins (Data Set S6).

10.1128/mSystems.00317-20.9 DATA SET S6 Selection criteria for single species sequencing and full DESeq2 results of transcript abundance analysis of *C. scindens* during dietary intervention and ciprofloxacin treatment. (Sheet 20) Total and average counts for metagenomic and metatranscriptomic read assignments generated via Kraken2. (Sheet 21) Differential abundance testing of the impact of Western diet (WD) consumption on the abundance of *C. scindens* transcripts within the murine cecal metatranscriptome. Log_2_ fold change values were calculated relative to control diet samples. (Sheet 22) Differential abundance testing of the impact of ciprofloxacin treatment on the abundance of *C. scindens* transcripts within the murine cecal metatranscriptome in animals consuming the Western diet (WD). Log_2_ fold change values were calculated relative to vehicle-treated samples on the WD. (Sheet 23) Differential abundance testing of the impact of ciprofloxacin treatment on the abundance of *C. scindens* transcripts within the murine cecal metatranscriptome in animals consuming the control diet (NC). Log_2_ fold change values were calculated relative to vehicle-treated samples on the NC. (Sheet 24) Interaction term analysis generated by DESeq2 for the impact of host diet consumption on changes in *C. scindens* transcript abundance following ciprofloxacin therapy. Log_2_ fold change values were calculated relative to vehicle-treated samples on the NC. Download Data Set S6, XLS file, 1.8 MB. Copyright © 2020 Cabral et al. 2020 Cabral et al. This content is distributed under the terms of the Creative Commons Attribution 4.0 International license.

**FIG 4:**
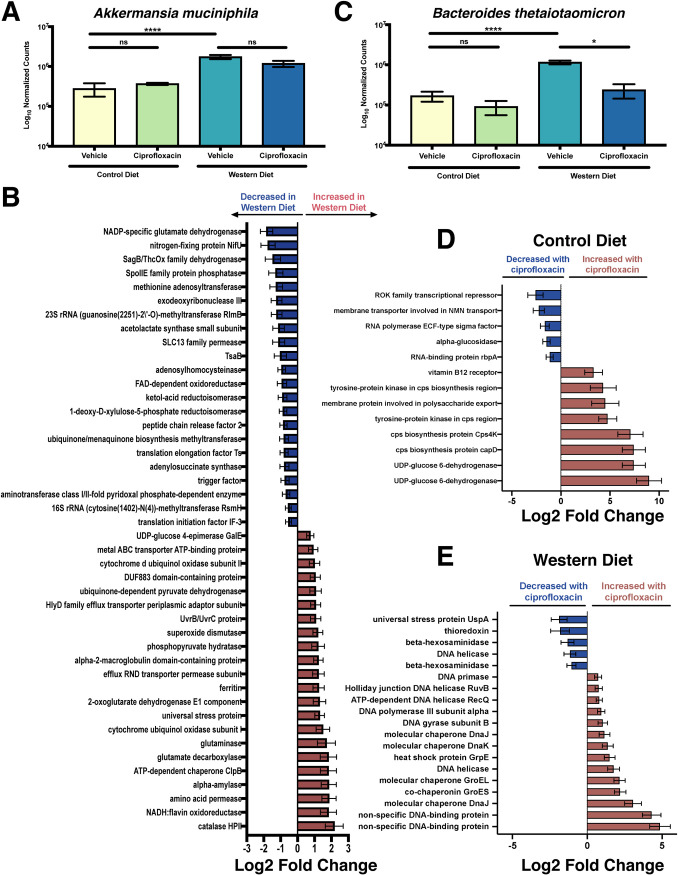
Diet and ciprofloxacin alter gene expression within B. thetaiotaomicron and *A. muciniphila*. (A) Normalized counts of *A. muciniphila* in each experimental group. Data are represented as mean ± SEM. Normalized counts were generated with DESeq2 and subsequently used to perform differential abundance testing. (*, *P* < 0.05; ****, *P* < 0.0001; Wald test with Benjamini and Hochberg correction). See Data Set S1 for full results. (B) Select differentially expressed (Benjamini-Hochberg-adjusted *P* value < 0.05) genes of interest in *A. muciniphila* within the cecum of vehicle-treated mice consuming the Western diet. Data are represented as log_2_ fold change relative to control diet ± standard error. See Data Set S7 for full results. (C) Normalized counts of B. thetaiotaomicron in each experimental group. Data are represented as mean ± SEM. Normalized counts were generated with DESeq2 and subsequently used to perform differential abundance testing. (*, *P* < 0.05; ****, *P* < 0.0001; Wald test with Benjamini and Hochberg correction). See Data Set S1 for full results. (D) Select differentially expressed (Benjamini-Hochberg-adjusted *P* value < 0.05) genes of interest in B. thetaiotaomicron within the cecum of ciprofloxacin-treated mice consuming the control diet. Data are represented as log_2_ fold change relative to vehicle-treated controls ± standard error. See Data Set S7 for full results. (E) Select differentially expressed (Benjamini-Hochberg-adjusted *P* value < 0.05) genes of interest in B. thetaiotaomicron within the cecum of ciprofloxacin-treated mice consuming the Western diet. Data are represented as log_2_ fold change relative to vehicle-treated controls ± standard error. See Data Set S7 for full results. For all analyses, *n* = 4. ns, not significant.

The Western diet significantly elevated the relative abundance of *A. muciniphila* (Fig. 4A). Interestingly, on this diet *A. muciniphila* displayed increased expression of several known stress response genes: catalase HPII, ATP-dependent chaperone ClpB, a universal stress protein, superoxide dismutase, and a UvrB/UvrC protein (Fig. 4B and Data Set S7). Additionally, we observed numerous changes in respiration and central carbon metabolism, including increased terminal oxidases, TCA cycle, glycolysis, and pyruvate metabolism, suggesting broad metabolic changes in response to the Western diet (Fig. 4B and Data Set 7). No CAZymes were differentially expressed on this diet, suggesting that the changes in *A. muciniphila* that facilitate its expansion are not driven by CAZyme activity (Data Set S7).

10.1128/mSystems.00317-20.10 DATA SET S7 Full DESeq2 results of transcript abundance analysis of *A. muciniphila* and B. thetaiotaomicron during dietary intervention and ciprofloxacin treatment and dietary formulation. (Sheet 25) Differential abundance testing of the impact of Western diet (WD) consumption on the abundance of *A. muciniphila* transcripts within the murine cecal metatranscriptome. Log_2_ fold change values were calculated relative to control diet samples. (Sheet 26) Differential abundance testing of the impact of ciprofloxacin treatment on the abundance of *A. muciniphila* transcripts within the murine cecal metatranscriptome in animals consuming the Western diet (WD). Log_2_ fold change values were calculated relative to vehicle-treated samples on the WD. (Sheet 27) Differential abundance testing of the impact of ciprofloxacin treatment on the abundance of *A. muciniphila* transcripts within the murine cecal metatranscriptome in animals consuming the control diet (NC). Log_2_ fold change values were calculated relative to vehicle-treated samples on the NC. (Sheet 28) Interaction term analysis generated by DESeq2 for the impact of host diet consumption on changes in *A. muciniphila* transcript abundance following ciprofloxacin therapy. Log_2_ fold change values were calculated relative to vehicle-treated samples on the NC. (Sheet 29) Differential abundance testing of the impact of Western diet (WD) consumption on the abundance of *A. muciniphila* CAZyme transcripts within the murine cecal metatranscriptome. Log_2_ fold change values were calculated relative to control diet samples. (Sheet 30) Differential abundance testing of the impact of ciprofloxacin treatment on the abundance of *A. muciniphila* CAZyme transcripts within the murine cecal metatranscriptome in animals consuming the Western diet (WD). Log_2_ fold change values were calculated relative to vehicle-treated samples on the WD. (Sheet 31) Differential abundance testing of the impact of ciprofloxacin treatment on the abundance of *A. muciniphila* CAZyme transcripts within the murine cecal metatranscriptome in animals consuming the control diet (NC). Log_2_ fold change values were calculated relative to vehicle-treated samples on the NC. (Sheet 32) Interaction term analysis generated by DESeq2 for the impact of host diet consumption on changes in *A. muciniphila* CAZyme transcript abundance following ciprofloxacin therapy. Log_2_ fold change values were calculated relative to vehicle-treated samples on the NC. (Sheet 33) Differential abundance testing of the impact of Western diet (WD) consumption on the abundance of B. thetaiotaomicron transcripts within the murine cecal metatranscriptome. Log_2_ fold change values were calculated relative to control diet samples. (Sheet 34) Differential abundance testing of the impact of ciprofloxacin treatment on the abundance of B. thetaiotaomicron transcripts within the murine cecal metatranscriptome in animals consuming the Western diet (WD). Log_2_ fold change values were calculated relative to vehicle-treated samples on the WD. (Sheet 35) Differential abundance testing of the impact of ciprofloxacin treatment on the abundance of B. thetaiotaomicron transcripts within the murine cecal metatranscriptome in animals consuming the control diet (NC). Log_2_ fold change values were calculated relative to vehicle-treated samples on the NC. (Sheet 36) Interaction term analysis generated by DESeq2 for the impact of host diet consumption on changes in B. thetaiotaomicron transcript abundance following ciprofloxacin therapy. Log_2_ fold change values were calculated relative to vehicle-treated samples on the NC. (Sheet 37) Differential abundance testing of the impact of Western diet (WD) consumption on the abundance of B. thetaiotaomicron CAZyme transcripts within the murine cecal metatranscriptome. Log_2_ fold change values were calculated relative to control diet samples. (Sheet 38) Differential abundance testing of the impact of ciprofloxacin treatment on the abundance of B. thetaiotaomicron CAZyme transcripts within the murine cecal metatranscriptome in animals consuming the Western diet (WD). Log_2_ fold change values were calculated relative to vehicle-treated samples on the WD. (Sheet 39) Differential abundance testing of the impact of ciprofloxacin treatment on the abundance of B. thetaiotaomicron CAZyme transcripts within the murine cecal metatranscriptome in animals consuming the control diet (NC). Log_2_ fold change values were calculated relative to vehicle-treated samples on the NC. (Sheet 40) Interaction term analysis generated by DESeq2 for the impact of host diet consumption on changes in B. thetaiotaomicron CAZyme transcript abundance following ciprofloxacin therapy. Log_2_ fold change values were calculated relative to vehicle-treated samples on the NC. (Sheet 41) Catalog number, macronutrient breakdown, and ingredient formulation of both the Control Diet and Western Diet (Research Diets, Inc., New Brunswick, NJ) used in this study. Download Data Set S7, XLS file, 4.6 MB. Copyright © 2020 Cabral et al. 2020 Cabral et al. This content is distributed under the terms of the Creative Commons Attribution 4.0 International license.

Ciprofloxacin treatment had a relatively minor impact on *A. muciniphila* gene expression (Data Set S7), likely due to the relatively low impact on the relative abundance of *A. muciniphila* (Fig. 4A). In total, ciprofloxacin significantly altered the expression of 2 and 17 genes on the control and Western diets, respectively (Data Set S7). On the control diet, *A. muciniphila* increased the expression of the molecular chaperone protein DnaK, which is known to play a role in stress responses (81–84). On the Western diet, several genes related to tryptophan biosynthesis and metabolism were elevated following ciprofloxacin treatment; however, their biological significance is unclear at this time (Data Set S7). Additionally, ciprofloxacin induced the differential expression of a sole chitin or lysozyme glycoside hydrolase, and only on the control diet (Fig. S3F and Data Set S7). Lastly, an examination of the interaction between diet and ciprofloxacin treatment indicated that only three genes were significantly altered. Overall, these data suggest that diet does not have a major impact on the response of this bacterium to ciprofloxacin within the microbiome (Data Set 7).

In contrast to *A. muciniphila*, diet had a relatively minor impact on B. thetaiotaomicron gene expression while ciprofloxacin induced extensive changes. Of note, B. thetaiotaomicron bloomed in response to the Western diet and was significantly perturbed by ciprofloxacin on this diet but not on the control (Fig. 4C). In total, 42 genes were altered in B. thetaiotaomicron in response to Western diet consumption (Data Set S7). Of note, this diet increased the expression of an aminoglycoside efflux pump and a hemin receptor. However, more than half of the genes (52.4%) that changed in response to diet are of unknown function and are classified as “hypothetical proteins;” making interpretation difficult. Interestingly, B. thetaiotaomicron did not exhibit robust changes in CAZyme transcription in response to the Western diet. Like *A. muciniphila*, B. thetaiotaomicron did not exhibit any differentially abundant CAZymes, suggesting that carbohydrate utilization does not drive the diet-induced changes in B. thetaiotaomicron abundance (Data Set S7). Ultimately, a description of this change will be dependent on improved functional annotations going forward.

On the control diet, we observed an increased abundance of transcripts encoding proteins involved in capsular polysaccharide (CPS) biosynthesis and export (Fig. 4D and Data Set S7). Within B. thetaiotaomicron, CPS production is encoded by a total of 182 genes distributed among eight loci (typically termed *cps1* to -*8*) (85, 86). It is hypothesized that an individual bacterium expresses one of these CPS configurations at any given time and that these structures play key roles in processes such as nutrient acquisition and immune evasion (86). Additionally, the two genes with the greatest increase in expression during ciprofloxacin treatment encoded UDP-glucose 6-dehydrogenase, which plays a key role in the biosynthesis of glycan precursors that are essential for capsule production in other bacteria (87–89). Together, these findings may suggest a role for CPS state as a determinant of ciprofloxacin susceptibility *in vivo*.

On the Western diet, ciprofloxacin elicited profound changes in transcriptional activity, altering the expression of 278 different genes (Fig. 4E and Data Set S7), and this robust response may be related to the reduction in B. thetaiotaomicron under this condition (Fig. 4C). Interestingly, expression of many genes involved in the utilization of host-derived carbohydrates (sialic acid-specific 9-*O*-acetylesterase, endo-beta-*N*-acetylglucosaminidase F1, beta-hexosaminidase) and stress responses (universal stress protein UspA, thioredoxin) was reduced, mirroring changes seen at the whole-community level (Fig. 4E and Data Set S7) in response to ciprofloxacin. Conversely, we observed increased expression of several genes that encode molecular chaperones or are involved in DNA replication or damage repair (Fig. 4E and Data Set S7). Ciprofloxacin triggers DNA damage via inhibition of DNA gyrase and topoisomerase IV. Thus, these changes in gene expression may be reflective of the primary mechanism of action of this antibiotic, are consistent with previously published data, and serve as a validation for our analysis (19).

Diet appears to have a significant impact on ciprofloxacin-induced transcriptional changes in B. thetaiotaomicron, modulating the response of 71 genes (Data Set S7). Of note, Western diet consumption in the context of ciprofloxacin treatment had a negative impact on several genes involved in the acquisition of nutrients, such as vitamin B_12_ and hemin receptors, and transporters of glucose/galactose, hexuronate, arabinose, and Na^+^ (Data Set S7). Thus, it is likely that the availability of nutrients within the gut plays a role in the response of these bacteria to antibiotics. Lastly, we examined the impact that nutrient availability has on the response of B. thetaiotaomicron CAZyme abundance to ciprofloxacin. We observed notable differences in CAZyme levels between the diets as well as differences in substrate targets (Fig. S3D and E and Data Set S7). On the control diet, B. thetaiotaomicron exhibits an increase in polysaccharide CAZymes, including those targeting pectin, rhamnogalacturonan, β-glucans, and hemicelluloses, with a simultaneous decrease in β-fucosidases (Fig. S3D and Data Set S7). On the Western diet, B. thetaiotaomicron exhibits an increase in lipopolysaccharide (LPS) biosynthesis and heparan degradation (Fig. S3E and Data Set S7). While interesting, more work will be required to elucidate mechanisms driving these phenotypes.

## DISCUSSION

Previous work has demonstrated that host diet, particularly with respect to sugar and fiber content, plays a major role in antibiotic-induced microbiome disruption (19, 30). In Western societies, many people consume a diet high in added sugars and fat but low in host-indigestible fiber. Such a composition is thought to promote the development of metabolic syndrome, heart disease, diabetes, and a number of other chronic conditions (36–46). Furthermore, broad-spectrum antibiotic use and resulting microbiome dysbiosis have been associated with a number of similar comorbidities along with increased susceptibility to opportunistic infections (1, 4, 5, 17, 20–22, 24, 25). Despite this connection, little work has been done examining how host dietary composition impacts the response of the microbiota to antibiotic perturbation. Nutrient availability and metabolic state are known to be major determinants of antibiotic susceptibility of bacteria *in vitro* (19, 27–29, 75, 90–95). Thus, modulating diet and subsequently nutrient availability to the microbiota would likely alter the sensitivity of bacteria in these communities to antibiotic therapy.

Using a combined metagenomic and metatranscriptomic approach, we demonstrate that diet composition has a major impact on the response of the murine gut microbiome to ciprofloxacin therapy. By utilizing these tools in parallel, we are able to link transcriptional changes to observed shifts in community structure on each diet. Using metagenomics, we observed that ciprofloxacin had a differential impact on community composition in a diet-dependent manner. Specifically, we observed a significant expansion of the *Firmicutes* phylum following ciprofloxacin treatment only on the Western diet. Metatranscriptomic data showed decreased abundance of transcripts from the TCA cycle after antibiotic treatment in both diets, suggesting that this response is diet independent, which is consistent with previous *in vitro* findings that demonstrate a key role for bacterial respiration as a determinant of fluoroquinolone susceptibility (27, 28, 77, 94, 96–98). Conversely, the impact of ciprofloxacin on the abundance of various iron and mucin utilization transcripts differed between diets. Lastly, we detected species-specific transcriptional changes in two important commensal bacteria, B. thetaiotaomicron and *A. muciniphila*. In addition to detecting changes in transcript levels that were reflective of stress responses, we also observed differential expression in transcripts involved in diverse cellular processes such as nutrient acquisition, carbon metabolism, and capsular polysaccharide (CPS) biosynthesis. Together, our findings supported our hypothesis that the Western diet would modify the metabolic capacity of the gut microbiome and that this change would directly translate to differential activity in response to ciprofloxacin treatment.

Despite the advantages of a multi-omic approach, there are several drawbacks to these techniques that complicate interpretation of the results. First, our study was performed only in female mice. It is now understood that sex-dependent differences exist in diet metabolism, mucosal immunity, and gut microbiome antibiotic responses, and as such our findings may not be generalizable to males (96, 99, 100). Another critical drawback is that the analytical pipelines used to analyze microbiome data are reliant on existing databases that are largely incomplete: approximately half of all genes within the human gut microbiome are hypothesized to have no functional annotation, limiting the ability to accurately profile the transcriptional activity of these communities (101). Additionally, inferring biological significance of taxonomic changes is often difficult in many microbiome analyses. 16S amplicon sequencing and shotgun metagenomics are inherently limited to reporting relative abundances and thus may fail to fully characterize changes in absolute abundance. Thus, we cannot comment on how diet or antibiotics change the total number of bacteria found in the gut, nor can we determine if the bloom in *Firmicutes* is a result of an increase in colony-forming units or a reduction of other bacteria relative to *Firmicutes*. Due to the complex nature of these communities, it is challenging to ascertain if the observed transcriptional changes are the result of the direct action of the antibiotic or the indirect effect of changes in host physiology, nutrient availability, or the disruption of ecological networks within the microbiome. For example, our transcriptional analysis of B. thetaiotaomicron showed that this bacterium differentially expressed receptors for both hemin and vitamin B_12_, which may suggest that these nutrients play a role in ciprofloxacin toxicity. Alternatively, it is possible that these transcriptional changes are reflective of increased availability of these nutrients due to decreased competition from other members of the microbiota. Further, dietary composition could play a significant role in antibiotic absorption or sequestration in the gut, which in turn would impact the extent of the damage caused to the microbiota.

This study builds on recent work that demonstrates that the availability of metabolites plays an important role in determining the extent of antibiotic-induced microbiome disruption (19). Taken together, these results demonstrate the need to consider dietary composition in the design and interpretation of experiments focused on understanding the impact of antibiotics on the microbiota. Previous studies have demonstrated that dietary changes induce rapid shifts in gut microbiome composition (32, 34, 43, 56, 97, 98, 102, 103). Therefore, in the long term, dietary modulation could represent an attractive strategy to reduce the collateral damage to commensal bacteria and the resulting complications from dysbiosis caused by clinical therapy. Despite these promising applications, considerable work is required before these findings have direct clinical relevance. In particular, the considerable differences in physiology, microbiome composition, and diet between humans and rodents complicate the direct clinical relevance of these findings. Furthermore, it is unclear whether short-term dietary modulation has any long-term consequences on either the host or the microbiome. Thus, additional research is warranted to fully elucidate how host diet impacts antibiotic-induced microbiome disruption in humans and how specific dietary formulation will impact these disruptions.

## MATERIALS AND METHODS

### Animal procedures.

All animal work was approved by Brown University’s Institutional Animal Care and Use Committee (IACUC) under protocol number 1706000283. Four-week-old female C57BL/6J mice were purchased from Jackson Laboratories (Bar Harbor, ME, USA) and given a 2-week habituation period immediately following arrival at Brown University’s Animal Care Facility. After habituation, mice were switched from standard chow (Laboratory Rodent Diet 5001; St. Louis, MO, USA) to either a Western diet (D12079B; Research Diets Inc., New Brunswick, NJ, USA) or a macronutrient-defined control diet (D12450B; Research Diets Inc., New Brunswick, NJ, USA) for 1 week (see Data Set S7, Sheet 41, in the supplemental material). On the 8th day of dietary intervention, mice were given acidified ciprofloxacin (12.5 mg/kg of body weight/day), or a pH-adjusted vehicle, via filter-sterilized drinking water *ad libitum* for 24 h (*n* = 8 to 12 per treatment group). Water consumption was monitored to ensure equal consumption across cages. Mice were then sacrificed and dissected in order to collect cecal contents. Cecal contents were immediately transferred to ZymoBIOMICS DNA/RNA Miniprep kit (Zymo Research, Irvine, CA, USA) collection tubes containing DNA/RNA Shield. Tubes were processed via vortexing at maximum speed for 5 min to homogenize cecal contents and then placed on ice until permanent storage at −80°C.

### Nucleic acid extraction and purification.

Total nucleic acids (DNA and RNA) were extracted from samples using the ZymoBIOMICS DNA/RNA Miniprep kit from Zymo Research (R2002; Irvine, CA, USA) using the parallel extraction protocol per the manufacturer’s instructions. Total RNA and DNA were eluted in nuclease-free water and quantified using the dsDNA-HS and RNA-HS kits on a Qubit 3.0 fluorometer (Thermo Fisher Scientific, Waltham, MA, USA) before use in library preparations.

### 16S rRNA amplicon preparation and sequencing.

The 16S rRNA V4 hypervariable region was amplified from total DNA using the barcoded 518F forward primer and the 816Rb reverse primers from the Earth Microbiome Project (104). Amplicons were generated using 5× Phusion high-fidelity DNA polymerase under the following cycling conditions: initial denaturation at 98°C for 30 s, followed by 25 cycles of 98°C for 10 s, 57°C for 30 s, and 72°C for 30 s, and then a final extension at 72°C for 5 min. After amplification, samples were pooled in equimolar amounts and visualized via gel electrophoresis. The pooled amplicon library was submitted to the Rhode Island Genomics and Sequencing Center at the University of Rhode Island (Kingston, RI, USA) for sequencing on the Illumina MiSeq platform. Amplicons were pair-end sequenced (2 × 250 bp) using the 500-cycle kit with standard protocols. We obtained an average of 106,135 ± 49,789 reads per sample.

### Analysis of 16S rRNA sequencing reads.

Raw 16S rRNA reads were subjected to quality filtering, trimming, denoising, and merging using the DADA2 package (version 1.8.0) in R (version 3.5.0). Ribosomal sequence variants were assigned taxonomy using the RDP Classifier algorithm with RDP Training set 16 using the *assignTaxonomy* function in DADA2 (105). Alpha diversity (Shannon) and beta diversity (Bray-Curtis dissimilarity) were calculated using the phyloseq package (version 1.24.2) in R (version 3.5.0).

### Metagenomic and metatranscriptomic library preparation.

Metagenomic libraries were prepared from DNA (100 ng) using the NEBNext Ultra II FS DNA library prep kit (New England BioLabs, Ipswich, MA, USA) >100-ng input protocol per the manufacturer’s instructions. This yielded a pool of 200- to 1,000-bp fragments where the average library was 250 to 500 bp. Metatranscriptomic libraries were prepared from total RNA using the NEBNext Ultra II Directional RNA sequencing prep kit (New England BioLabs, Ipswich, MA, USA) in conjunction with the NEBNext rRNA depletion kit for human/mouse/rat (New England BioLabs, Ipswich, MA, USA) and the MICROBExpress kit (Invitrogen, Carlsbad, CA, USA). First, up to 1 μg of total RNA was treated with recombinant DNase I (rDNase I) and subsequently depleted of bacterial rRNAs using MICROBExpress per the manufacturer’s instructions. This depleted RNA was then used to prepare libraries with the NEBNext Ultra II Directional RNA sequencing prep and rRNA depletion kits per the manufacturer’s instructions. This yielded libraries that averaged between 200 and 450 bp. Once library preparation was complete, both metagenomic and metatranscriptomic libraries were sequenced as paired-end 150-bp reads on an Illumina HiSeq X Ten. We sequenced an average of 2,278,948,631 (±2,309,494,556) bases per metagenomic sample and 14,751,606,319 (±3,089,205,166) bases per metatranscriptomic sample. One metagenomic sample from the Western diet + vehicle group had an abnormally low number of bases sequenced (165,000 bp) and was excluded from all subsequent analyses. Following the removal of this sample, we obtained an average of 2,430,867,540 (±2,306,317,898) bases per metagenomic sample.

### Processing of raw metagenomic and metatranscriptomic reads.

Raw metagenomic reads were trimmed and decontaminated using the kneaddata utility (version 0.6.1) (106). In brief, reads were first trimmed to remove low-quality bases and Illumina TruSeq3 adapter sequences using Trimmomatic (version 0.36) using a SLIDINGWINDOW value of 4:20 and an ILLUMINACLIP value of 2:20:10, respectively (107). Trimmed reads shorter than 75 bases were discarded. Reads passing quality control were subsequently decontaminated by removing those that mapped to the genome of C57BL/6J mice using bowtie2 (version 2.2) (108). Additionally, preliminary work by our group detected high levels of reads mapping to two murine retroviruses found in our animal facility: murine mammary tumor virus (MMTV, accession NC_001503) and murine osteosarcoma viruses (MOV, accession NC_001506.1) (19). Raw metatranscriptomic reads were trimmed and decontaminated using the same parameters. However, in addition to removing reads that mapped to the C57BL/6J, MMTV, and MOV genomes, we also decontaminated sequences that aligned to the SILVA 128 LSU and SSU Parc rRNA databases (109).

### Taxonomic classification of metagenomic reads.

Trimmed and decontaminated metagenomic reads were taxonomically classified against a database containing all bacterial and archaeal genomes found in NCBI RefSeq using Kraken2 (version 2.0.7-beta) with a default k-mer length of 35 (110). Phylum- and species-level abundances were subsequently calculated from Kraken2 reports using Bracken (version 2.0.0) with default settings (111). The phyloseq package (version 1.28.0) in R (version 3.6.0) was used to calculate alpha diversity using the Shannon diversity index (112). Metagenomic data were not subsampled prior to analysis.

To perform differential abundance testing, species-level taxonomic output was first filtered to remove taxa that were not observed in >1,000 reads (corresponding to approximately 0.1% of all reads) in at least 20% of all samples using phyloseq in R. Differential abundance testing was subsequently performed on filtered counts using the DESeq2 package (version 1.24.0) using default parameters (74). All *P* values were corrected for multiple hypothesis testing using the Benjamini-Hochberg method (113).

### Annotation of metatranscriptomic reads using SAMSA2.

Trimmed and decontaminated metatranscriptomic reads were annotated using a modified version of the Simple Annotation of Metatranscriptomes by Sequence Analysis 2 (SAMSA2) pipeline as described previously (19, 63, 114). First, the Paired-End Read Merger (PEAR) utility was used to merge forward and reverse reads (115). Merged reads were then aligned to databases containing entries from the RefSeq, SEED Subsystems, and CAZyme databases using DIAMOND (version 0.9.12) (116–118). The resulting alignment counts were subsequently analyzed using DESeq2 (version 1.24.0) using the Benjamini-Hochberg method to perform multiple hypothesis testing correction (19, 63, 113). Features with an adjusted *P* value of less than 0.05 were considered to be statistically significant.

### Metatranscriptomic analysis using HUMAnN2.

To determine the impact of dietary modulation and ciprofloxacin treatment on gene expression within the gut microbiome, we used the HMP Unified Metabolic Analysis Network 2 (HUMAnN2, version 0.11.1) pipeline (64). First, metagenomic reads were taxonomically annotated using MetaPhlan2 (version 2.6.0) and functionally annotated against the UniRef90 database to generate gene family and MetaCyc pathway-level abundances. To ensure consistent assignment between paired samples, the taxonomic profile generated from the metagenomic reads was supplied to the HUMAnN2 algorithm during the analysis of the corresponding metatranscriptomic reads. Metatranscriptomic reads were subsequently annotated as done for metagenomic reads. The resulting gene family and pathway-level abundance data from the metatranscriptomic reads were normalized against the metagenomic data from the corresponding sample and smoothed using the Witten-Bell method (119). Lastly, the resulting RPKM (reads per kilobase per million) values were unstratified to obtain whole-community level data, converted into relative abundances, and analyzed using LEfSe (version 1) hosted on the Galaxy web server (120).

### Transcriptional analysis of *A. muciniphila* and B. thetaiotaomicron.

A modified version of a previously published pipeline from Deng et al. was utilized to perform transcriptional analysis of individual species within the murine microbiome during dietary modulation and antibiotic treatment (19, 80). First, Kraken2 (version 2.0.7-beta) was used to identify the 50 most prevalent bacterial species present within the metatranscriptomic samples (110). Next, the BBSplit utility within the BBMap package (version 37.96) was used to extract reads within our metatranscriptomic data set that mapped to these 50 most abundant species (121). Reads from B. thetaiotaomicron, *A. muciniphila*, and *C. scindens* were subsequently aligned to their corresponding reference genomes using the BWA-MEM algorithm (version 0.7.15) (122). Lastly, the featureCounts command within the subread program (version 1.6.2) was used to analyze the resulting alignment files to generate a count table for differential expression analysis with DESeq2 (74). All *P* values were corrected for multiple hypothesis testing with the Benjamini-Hochberg method (113). Features with an adjusted *P* value of less than 0.05 were considered to be statistically significant.

### Data availability.

The data sets generated and analyzed during this study are available from the NCBI Sequence Read Archive (SRA) under BioProject accession numbers PRJNA563913 (metagenomics and metatranscriptomics) and PRJNA594642 (16S rRNA amplicon sequences). Any additional information is available from the corresponding author upon request.
