# Supplementary figures and images for: Consumption of a Western-Style Diet Modulates the Response of the Murine Gut Microbiome to Ciprofloxacin
Source: mSystems. 2020 Jul 28;5(4):e00317-20. doi: 10.1128/mSystems.00317-20 (PMC7394352; doi:10.1128/mSystems.00317-20)

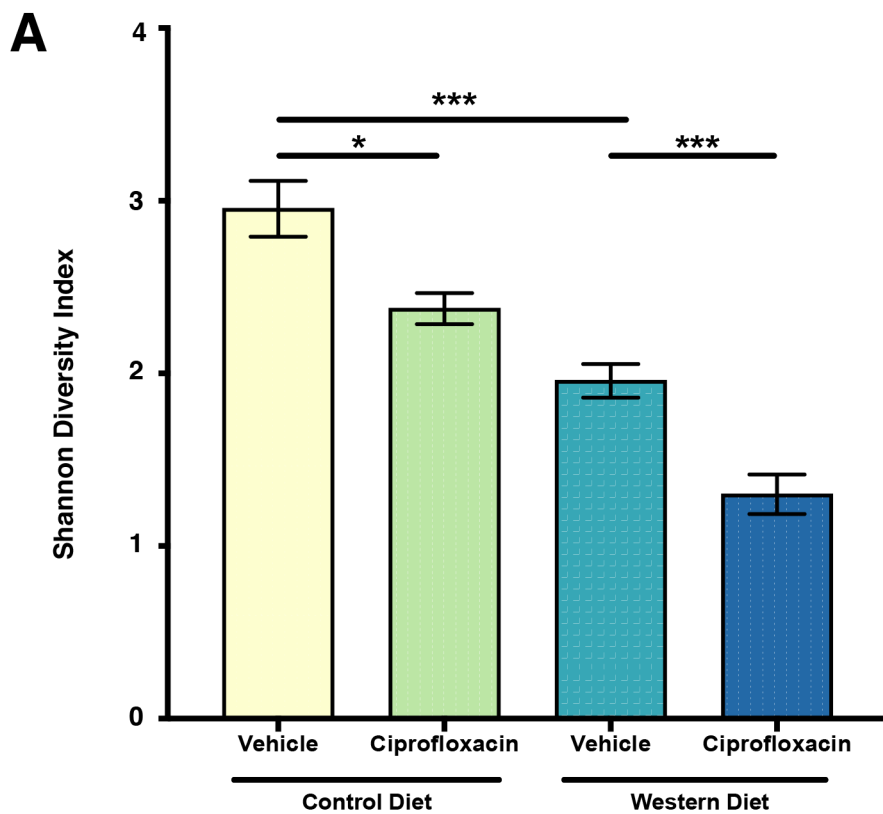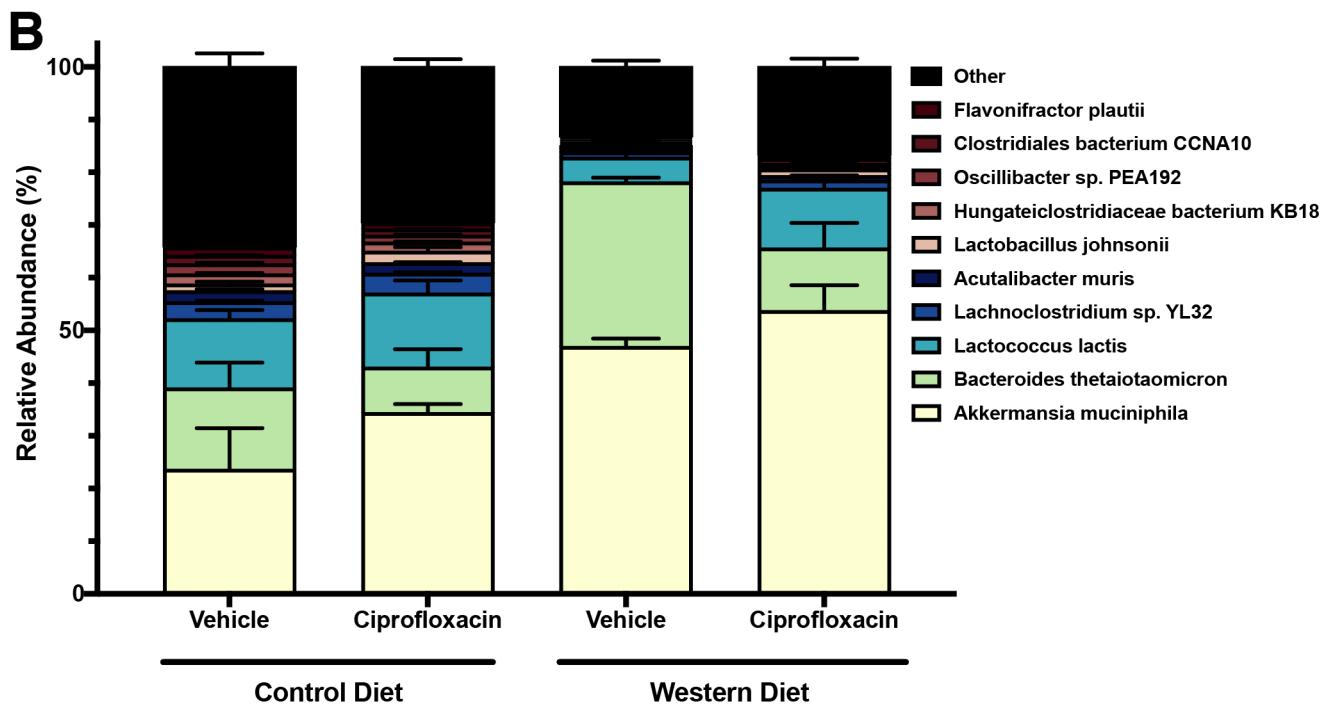

Supplement: FIG S1 [file mSystems.00317-20-sf001.pdf]

**A**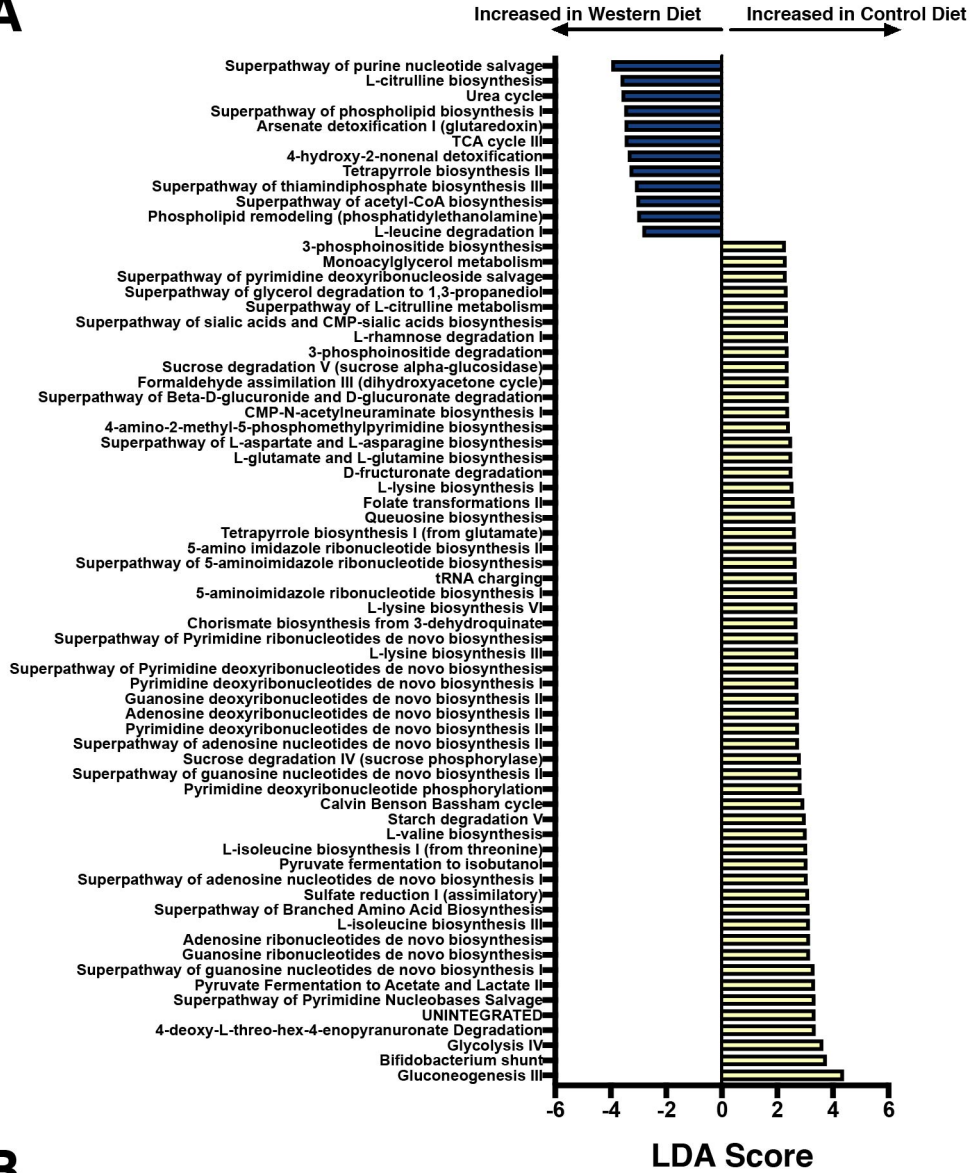**B**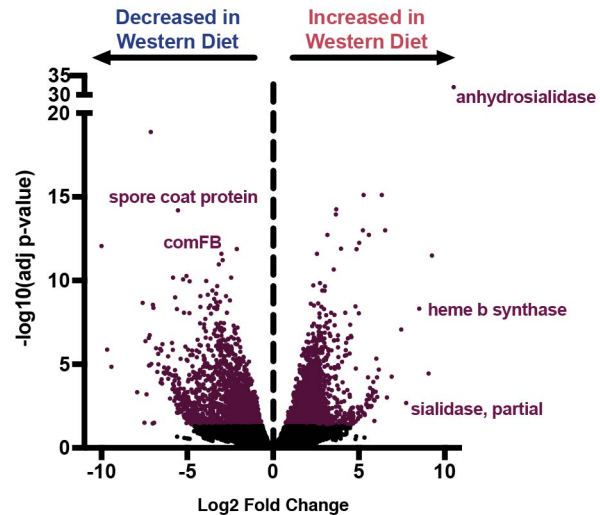

Supplement: FIG S2 [file mSystems.00317-20-sf002.pdf]
