# Supplementary material for: Consumption of a Western-Style Diet Modulates the Response of the Murine Gut Microbiome to Ciprofloxacin
Source: mSystems. 2020 Jul 28;5(4):e00317-20. doi: 10.1128/mSystems.00317-20 (PMC7394352; doi:10.1128/mSystems.00317-20)
Supplement: FIG S3 [file mSystems.00317-20-sf003.pdf]

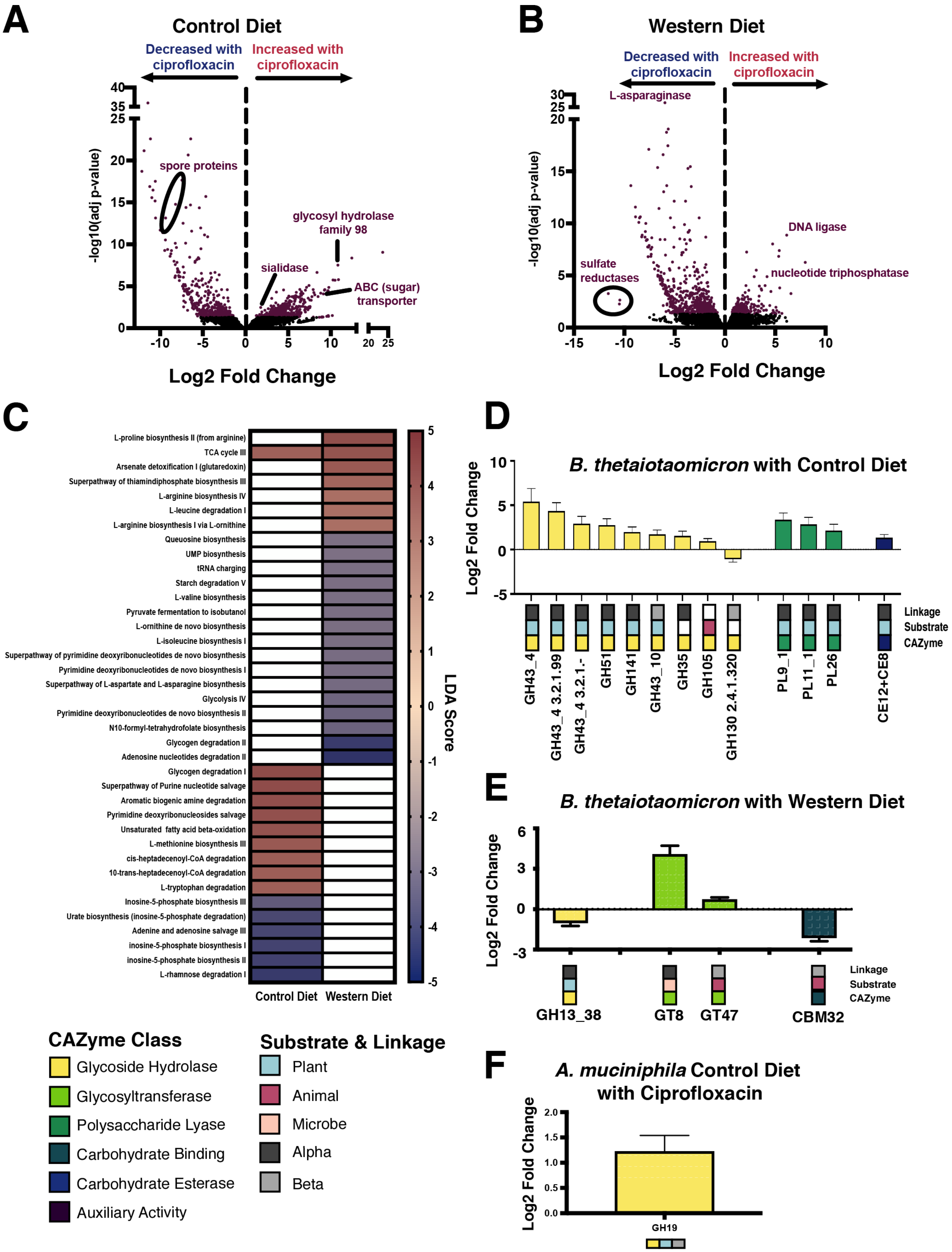

A
Control Diet
Decreased with ciprofloxacin
Increased with ciprofloxacin
spore proteins
sialidase
glycosyl hydrolase family 98
ABC (sugar) transporter
-log10(adj p-value)
Log2 Fold Change
B
Western Diet
Decreased with ciprofloxacin
Increased with ciprofloxacin
L-asparaginase
sulfate reductases
DNA ligase
nucleotide triphosphatase
-log10(adj p-value)
Log2 Fold Change
C
L-proline biosynthesis II (from arginine)
TCA cycle III
Arsenate detoxification I (glutaredoxin)
Superpathway of thiamindiphosphate biosynthesis III
L-arginine biosynthesis IV
L-leucine degradation I
L-arginine biosynthesis I via L-ornithine
Queuosine biosynthesis
UMP biosynthesis
tRNA charging
Starch degradation V
L-valine biosynthesis
Pyruvate fermentation to isobutanol
L-ornithine de novo biosynthesis
L-isoleucine biosynthesis I
Superpathway of pyrimidine deoxyribonucleotides de novo biosynthesis
Pyrimidine deoxyribonucleotides de novo biosynthesis I
Superpathway of L-aspartate and L-asparagine biosynthesis
Glycolysis IV
Pyrimidine deoxyribonucleotides de novo biosynthesis II
N10-formyl-tetrahydrofolate biosynthesis
Glycogen degradation II
Adenosine nucleotides degradation II
Glycogen degradation I
Superpathway of Purine nucleotide salvage
Aromatic biogenic amine degradation
Pyrimidine deoxyribonucleosides salvage
Unsaturated fatty acid beta-oxidation
L-methionine biosynthesis III
cis-heptadecenoyl-CoA degradation
10-trans-heptadecenoyl-CoA degradation
L-tryptophan degradation
Inosine-5-phosphate biosynthesis III
Urate biosynthesis (inosine-5-phosphate degradation)
Adenine and adenosine salvage III
inosine-5-phosphate biosynthesis I
inosine-5-phosphate biosynthesis II
L-rhamnose degradation I
Control Diet
Western Diet
LDA Score
D
B. thetaiotaomicron with Control Diet
Log2 Fold Change
Linkage
Substrate
CAZyme
GH43_4
GH43_4 3.2.1.99
GH43_4 3.2.1.-
GH51
GH141
GH43_10
GH35
GH105
GH130 2.4.1.320
PL9_1
PL11_1
PL26
CE12+CE8
E
B. thetaiotaomicron with Western Diet
Log2 Fold Change
Linkage
Substrate
CAZyme
GH13_38
GT8
GT47
CBM32
CAZyme Class
Glycoside Hydrolase
Glycosyltransferase
Polysaccharide Lyase
Carbohydrate Binding
Carbohydrate Esterase
Auxiliary Activity
Substrate & Linkage
Plant
Animal
Microbe
Alpha
Beta
F
A. muciniphila Control Diet with Ciprofloxacin
Log2 Fold Change
GH19
